# Merkel cell carcinoma originating in the gluteal region

**DOI:** 10.1016/j.jdcr.2025.04.030

**Published:** 2025-05-21

**Authors:** Shlomit Fennig, Yosef Landman, Ronen Brenner, Eyal Fenig

**Affiliations:** a Institute of Oncology, Kaplan Medical Center, Rehovot, Israel; b Faculty of Medicine, Hebrew University, Jerusalem, Israel; c Institute of Oncology, Davidoff Cancer Center, Rabin Medical Center – Beilinson Hospital, Petach Tikva, Israel; d Faculty of Medicine, Tel Aviv University, Tel Aviv, Israel; e Institute of Oncology, Wolfson Medical Center, Holon, Israel

**Keywords:** chemotherapy, gluteal region, immunotherapy, Merkel cell carcinoma, radiation therapy

## Introduction

Merkel cell carcinoma (MCC) is a rare neuroendocrine tumor of the skin. It is highly aggressive, with a mortality rate of 33% in 5 years.^1^ Risk factors contributing to MCC development include those over the age of 65 yearsears, fair skin, exposure to UV radiation, infection with Merkel cell polyomavirus, and chronic immunosuppression.^2^, ^3^, ^4^, ^5^, ^6^, ^7^ MCC originating in non-sun-exposed skin, particularly the gluteal region, is extremely rare, with only a few cases reported in the literature.^8^, ^9^, ^10^

The aim of this study was to present, for the first time, the demographics and clinical outcomes of the largest reported cohort of patients with MCC that originated in the gluteal region.

## Methods

### Patients and setting

A retrospective study was conducted using the electronic health care database of a tertiary university-affiliated medical center. Patients diagnosed with MCC between 1984 and 2024 were identified, and those with MCC originating in the gluteal region were selected. Their demographic and clinical data were collected from the medical files.

### Statistical analysis

Overall survival (OS), progression-free survival, and disease-specific survival were calculated from the date of diagnosis to the date of last follow-up or death and predicted with the Kaplan-Meier method. All statistical analyses were performed using SPSS software (IBM Corp., 2017. IBM SPSS Statistics for Windows).

## Results

A total of 271 patients were diagnosed with MCC during the study period, of whom 39 (14.4%) had regional lymph node metastases with no detectable primary cutaneous tumor. The remaining 232 patients (85.6%) had a clearly defined primary of cutaneous origin, including 25 (10.8%) with a primary tumor located in the gluteal region. Other sites included the limbs in 104 patients (44.8%), head and neck in 85 (36.6%) patients, and torso in 18 (7.8%) patients.

The demographic and clinical characteristics of the 25 patients with MCC originating in the gluteal skin are shown in Table I. The median age was 65 years (range: 47-91 years; mean ± SD: 69 ± 12.2 years), and the male-to-female ratio was 3:2. Most patients had advanced disease at diagnosis: 12 (48%) with regional lymph node metastases (stage IIIA-B) and 3 (12%) with distant metastases (stage IV). The remainder presented with stage I (4 patients) or stage IIA-B (6 patients). Treatment of all patients with stage II disease consisted of surgery followed by radiotherapy to the tumor bed and groin lymph nodes. Among the patients with stage III disease, those with resectable tumors (*n* = 6) were treated with surgery and adjuvant chemoradiation, including cisplatin or carboplatin with etoposide; those with unresectable disease (*n* = 6) received definitive chemoradiation at a higher dose of 60 Gy. 9 patients, 6 with unresectable stage III MCC and 3 with metastatic stage IV, underwent diagnostic biopsy. One patient with stage I disease underwent excisional biopsy with curative intent.

**Table I:** Patient and treatment characteristics (*N* = 25)

Variable	Value
Age (y), median [range]	65 [47-91]
Sex	
Male	15 (60%)
Female	10 (40%)
Side	
Right	16 (64%)
Left	9 (36%)
Stage	
I	4 (16%)
IIA	5 (20%)
IIB	1 (4%)
IIIA	1 (4%)
IIIB	11 (44%)
IV	3 (12%)
Surgery type	
WE + SLNB	13 (52%)
WE + LND	2 (8%)
Biopsy only	10 (40%)
Concurrent chemotherapy	
Yes	12 (48%)
No	13 (52%)
Best response	
Complete	19 (76%)
Partial	3 (12%)
Partial disease	3 (12%)

Values are presented as *n* (%) unless otherwise indicated.

*LND*, Lymph node dissection; *SLNB*, sentinel lymph node biopsy; *WE*, wide excision.

**Fig 1:**
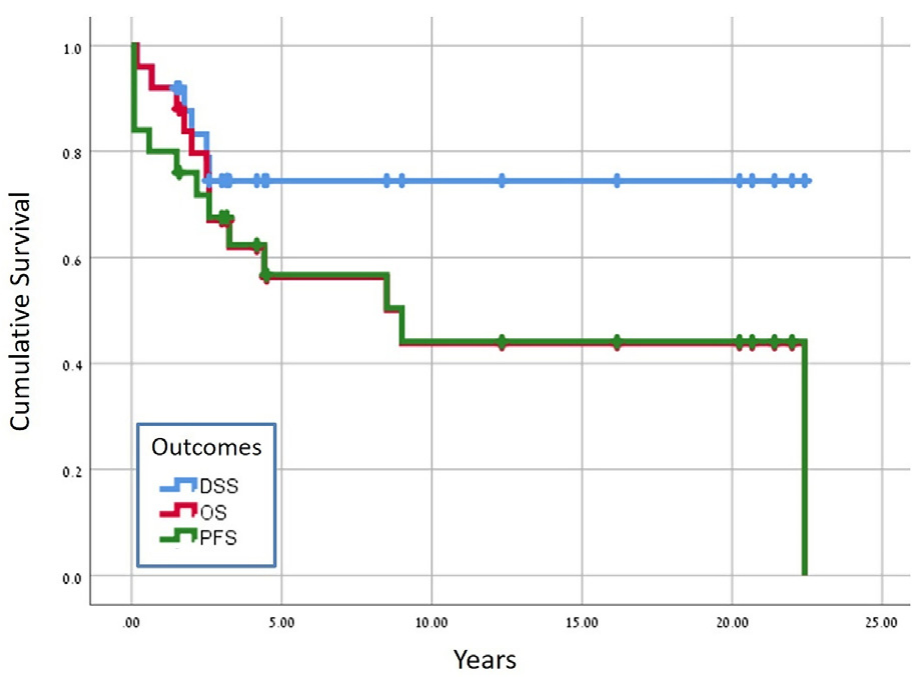
Survival outcome for gluteal MCC. *DSS*, Disease-specific survival; *MCC*, merkel cell carcinoma; *OS*, overall survival; *PFS*, progression-free survival.

The survival data are shown in Fig 1. Of the 6 patients with unresectable stage III MCC, 1 died of the disease at 21 months and 5 were alive and disease-free for 38 to 257 months. Of the 3 patients with metastatic stage IV MCC, 2 died of the disease at 7 months and 1 was cured of disease and died at 257 months of another cause.

The median duration of follow-up for the whole cohort was 50 months (range: 2-264 months), and for the 12 surviving patients, 100 months (range: 18-264 months). The 5-year OS was 60.8%, progression-free survival 56.7%, and disease-specific survival 78.4%. The corresponding 10-year values were 54.1%, 44.1%, and 78.4%.

## Discussion

This is the first comprehensive study in the literature of the clinical and demographic characteristics of a relatively large group of patients with MCC of primary gluteal origin. Only a few case reports of gluteal MCC have been published to date.^8^, ^9^, ^10^

The findings in our cohort differ from earlier studies of MCC based on the Surveillance, Epidemiology, and End Results Program database^3^ in terms of mean age (69 vs 74 years) and male-to-female ratio (3:2 vs 2.2:1). In an earlier study of 9387 cases of MCC derived from the National Cancer Database, 65% of patients presented with local disease (stages 1/11), whereas the rate in our cohort was only 40%. Nevertheless, the 60.8% 5-year OS in our cohort was considerably better than the reported estimates of 51%, 35%, and 14% for local, nodal, and distant disease, respectively.^11^ One explanation for this discrepancy is the better cure rate in our patients with locally advanced (stage III) disease, possibly owing to our aggressive treatment approach with adjuvant or definitive chemoradiation. In addition, tumors originating outside the head and neck area may be associated with better OS than MCC in other cutaneous sites because of different mechanisms of carcinogenesis with Merkel cell polyomavirus infection and less exposure to UV radiation.^12^ This suggestion is supported by a study of 976 cases of MCC of the lower limb and hip extracted from the Surveillance, Epidemiology, and End Results database^12^ and a smaller study (*n* = 27) comparing patients with head-and-neck and non-head-and-neck MCC.^13^ That the buttocks are by definition a low-UV-exposure region could account for the better prognosis in our patients as well.

In conclusion, the present pioneer study of MCC originating in the gluteal region in a relatively large cohort of patients identified several characteristics distinct from MCC originating in sun-exposed anatomic areas in the general population, including younger patient age, advanced disease at diagnosis, and better long-term prognosis. Further controlled studies are needed in larger cohorts from different geographic regions.

## Conflicts of interest

None disclosed.
